# From Cancer to Immune Organoids: Innovative Preclinical Models to Dissect the Crosstalk between Cancer Cells and the Tumor Microenvironment

**DOI:** 10.3390/ijms251910823

**Published:** 2024-10-09

**Authors:** Francesca Picca, Claudia Giannotta, Jiahao Tao, Lucia Giordanengo, H. M. Waqas Munir, Virginia Botta, Alessandra Merlini, Andrea Mogavero, Edoardo Garbo, Stefano Poletto, Paolo Bironzo, Gabriella Doronzo, Silvia Novello, Riccardo Taulli, Francesca Bersani

**Affiliations:** 1 Department of Oncology, University of Torino, S. Luigi Gonzaga Hospital, Regione Gonzole 10, 10043 Orbassano, Italy; 2 Molecular Biotechnology Center ‘Guido Tarone’, University of Torino, Piazza Nizza 44, 10126 Torino, Italy; 3 Department of Molecular Biotechnology and Health Sciences, University of Torino, Via Nizza 52, 10126 Torino, Italy; 4 Thoracic Unit and Medical Oncology Division, Department of Oncology, University of Torino, S. Luigi Gonzaga Hospital, 10043 Orbassano, Italy

**Keywords:** organotypic cultures, patient-derived tumor organoids (PDTOs), patient-derived tumor xenografts (PDTXs), immune organoids, tumor microenvironment, precision oncology

## Abstract

Genomic-oriented oncology has improved tumor classification, treatment options, and patient outcomes. However, genetic heterogeneity, tumor cell plasticity, and the ability of cancer cells to hijack the tumor microenvironment (TME) represent a major roadblock for cancer eradication. Recent biotechnological advances in organotypic cell cultures have revolutionized biomedical research, opening new avenues to explore the use of cancer organoids in functional precision oncology, especially when genomics alone is not a determinant. Here, we outline the potential and the limitations of tumor organoids in preclinical and translational studies with a particular focus on lung cancer pathogenesis, highlighting their relevance in predicting therapy response, evaluating treatment toxicity, and designing novel anticancer strategies. Furthermore, we describe innovative organotypic coculture systems to dissect the crosstalk with the TME and to test the efficacy of different immunotherapy approaches, including adoptive cell therapy. Finally, we discuss the potential clinical relevance of microfluidic mini-organ technology, capable of reproducing tumor vasculature and the dynamics of tumor initiation and progression, as well as immunomodulatory interactions among tumor organoids, cancer-associated fibroblasts (CAFs) and immune cells, paving the way for next-generation immune precision oncology.

## 1. Introduction

### 1.1. Preclinical Models in Translational Cancer Research

#### 1.1.1. Cell Lines and Mouse Models

Bidimensional cultures and genetically engineered mouse models are generally exploited to study different aspects of cancer pathogenesis such as tumor initiation, dissemination, and evolution [1,2]. However, several drawbacks have hampered their exploitation in personalized medicine. Accordingly, only limited anticancer drugs originally identified in pre-clinical models have been subsequently introduced into clinical practice [3]. Indeed, although commercial cell lines still constitute the most widely employed preclinical models for in vitro studies due to their easy growth and manipulation, they are subjected to genetic drifting, losing the original tumor mutational signature [4]. Patient-derived tumor xenografts (PDTXs), where tumor specimens are grown in immunodeficient recipient mice, represent robust preclinical platforms to maintain cancer heterogeneity and to monitor tumor evolution under therapeutic pressure [5]. PDTXs preserve the tridimensional tumor architecture and can shed light on functional interactions between the tumor and the extracellular matrix. However, human non-transformed stromal cells and tumor immune infiltrate are rapidly lost in PDTXs and establishment rates remain costly and time-consuming [6].

#### 1.1.2. Organotypic Cultures

Recent advances in culture growth conditions have allowed the in vitro generation of organotypic models, where tumor cells are embedded in a semi-solid matrix, and once stimulated with the appropriate cocktail of cytokines and growth factors, they can sustain the expansion of all the different cell populations characterizing the primitive tumor. Specifically, tridimensional tumor organoids consist of well-organized structures where stem, progenitor, and differentiated cells coexist in a condition that closely emulates the primitive neoplastic lesion [7]. Notably, the self-renewal hierarchical organization that drives organotypic growth is pivotal to maintain the original genetic heterogeneity in long-term stabilized cultures [8]. Indeed, the extraordinary potential of this approach is the possibility to preserve the original tumor features at both the genomic and functional level. In addition, although the generation of patient-derived tumor organoids (PDTOs) is still challenging for specific tumor types, they have raised a tremendous interest in the new field of personalized oncology, ranging from basic to translational studies [9]. Currently, the potential of PDTOs spans from advanced drug screening platforms [10] to the identification of novel actionable biomarkers for rare neoplasms [11] and sophisticated lineage tracing applications for the study of cell dormancy [12]. It is thus not surprising that the generation of in vitro organoids was recognized as the technology of the year already in 2017 [13].

### 1.2. Challenges in Precision Oncology

#### 1.2.1. State of the Art in Lung Cancer

**Figure 1:**
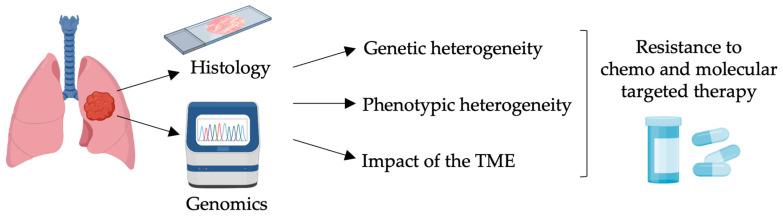
Advances and challenges in precision oncology. The implementation of next-generation sequencing with immunohistochemical analysis in the current decision-making process has improved patient stratification and outcomes, especially with the introduction of molecular targeted therapies in clinical practice. Emerging evidence highlights that therapy resistance is not only driven by genetics, but it also relies on cancer cell plasticity and on how cancer shapes the tumor microenvironment (TME). Therefore, innovative strategies are required for cancer eradication in a personalized manner. Created with BioRender.com.

Lung cancer is the leading cause of cancer death worldwide, with a mortality rate exceeding that of breast, colon, and prostate cancers [14]. According to the current WHO classification [15], lung cancer is classified into two major histological subtypes, namely non-small cell lung cancer (NSCLC) and small cell lung cancer (SCLC), accounting for 80–85% and 15–20% of cases, respectively. The major histological subtypes of NSCLC are represented by adenocarcinoma, squamous cell carcinoma, and large cell carcinoma, which occur with a frequency of 40%, 20–30%, and 15%, respectively [15]. Adenocarcinoma represents the predominant lung epithelial neoplasm in younger and non-smoking patients [16]. In contrast to other tumor types, the life expectancy for patients affected by lung tumors is still dismal, especially in advanced stages. Indeed, the 5-year overall survival rate is 19%, with a higher rate for NSCLC (23%) compared to SCLC (6%) [17]. At the clinical level, the cancer stage at the time of diagnosis determines the selection of subsequent therapeutic regimens. The gold standard for the management of early-stage and resectable locally advanced NSCLC is surgery, with the addition of neoadjuvant or adjuvant treatments based on the specific stage and molecular profile [18]. However, since symptoms typically occur in advanced stages, the majority of patients are diagnosed with distant metastases (stage IV), when surgical intervention is no longer an option [19]. Unfortunately, even patients with early-stage or locally advanced disease almost inevitably relapse. In this scenario, therapies are substantially not curative, and they are aimed at improving the overall survival and quality of life. The recent introduction of next-generation sequencing (NGS) into clinical practice has contributed to define the molecular profile of lung cancer, leading to the identification of several actionable targets amenable to targeted therapies and dramatically changing the therapeutic perspective for oncogene-addicted NSCLC patients, accounting for about two-thirds of the cases. The most common targetable driving mutations occur in *KRAS*, *EGFR*, *BRAF*, *MET*, *ALK*, *ERBB2*, *ROS1*, and *RET* oncogenes [20]. Nevertheless, the benefit of molecular therapies is temporally limited due to the development of resistance, which almost invariably ensues with targeted treatments [21,22]. Indeed, therapy evasion represents a major hurdle for cancer treatment [23] (Figure 1).

#### 1.2.2. Mechanisms of Therapy Evasion

Conceptually, intra- and inter-tumoral heterogeneity at the genetic level are considered the main cause of molecular targeted therapy failure, since rare mutated subclones can be selected under drug pressure and may thus rapidly emerge. The constant acquisition of a mutator phenotype is typical of lung cancer and in *ALK*-rearranged NSCLC cells, this process is accelerated by key drivers of mutagenesis such as APOBEC cytidine deaminases [24]. Following the classical Darwinian vision, both pre-existing and de novo acquired subclonal variabilities are responsible for treatment failure and patient relapse [25], but identification of these rare variants is challenging and cost-effective [26]. Moreover, recent evidence suggests that tumor cell plasticity, referred to also as non-genetic adaption to drug treatment, is critical for cancer cell survival and evolution in lung cancer [27,28,29]. Indeed, tumor cells can adopt multiple reversible phenotypic states, including dormancy and transcriptional fluctuations in response to therapy [30]. In this context, drug-tolerant persister cells are emerging as key players in guiding phenotypic heterogeneity and evolvability under therapeutic pressure thanks to their intrinsic plasticity at the genomic and functional level [31].

#### 1.2.3. Contribution of the Microenvironment

Tumor development and therapy resistance have been recently conceived in a broader vision that includes key principles of ecology [31,32].This concept implies the existence of a ‘cancer biosphere’ within the human body, where the capability of the ‘alien’ (the tumor) to grow, disseminate, and potentially resist to therapy is not merely associated with its own intrinsic genetic/epigenetic features, but it is also linked to the fruitful functional relationships established with normal cells that occupy the same “habitat” niche. These aspects are particularly relevant in all phases of tumorigenesis, from cancer initiation to dissemination, as well as during therapy-induced evolution. Indeed, mounting evidence suggests a continuous interplay between the tumor and the normal host environment, involving cells close to the tumor niche together with those localized in distal body districts [32]. Therefore, innovative anticancer strategies should be designed considering the impact of the tumor ecosystem in drug responses. Indeed, while the tumor shapes the normal ecosystem to support its own growth, it is now well established that the path toward resistance requires the support of the normal counterpart. Within this framework, recent improvements in tridimensional cultures and technological advances in biocompatible devices are available to deconstruct tumor complexity in vitro and to explore novel effective therapies for cancer cure, including immunotherapy.

## 2. Mimicking Lung Cancer with PDTOs

### 2.1. Overview of Existing Lung PDTO Platforms

#### 2.1.1. Establishment of Lung Cancer PDTO Cultures

The first robust protocol for the generation of organotypic cultures from bronchoalveolar tumor resections or pulmonary lavage was published in 2019 [33]. Notably, airway organoids preserved the genomic and histological characteristics of the matched patients and were suitable to perform drug screening in a personalized manner. Most importantly, Sachs et al. not only highlighted the potential of this technology for the improvement of lung cancer precision oncology, but also showed its translational relevance for infectious and genetic pulmonary diseases [33]. In the same year, a large collection of PDTOs from lung cancer patients and normal airway organoids was reported [34]. Normal bronchial organoids maintained their original epithelial hierarchical organization, and the 80 PDTOs originated from five different subtypes of lung cancer, faithfully recapitulated the original histological and genetic features of the patient tissue. Interestingly, this protocol achieved a 70% success rate in the generation of organoids, which also conserved PD-L1 expression, pinpointing the potential utility of this platform for the study of the tumor immune microenvironment (TME) in the context of immune checkpoint inhibition. Moreover, lung PDTOs preserved the tumorigenic potential, and they were exploitable for drug screening tests and for the generation of additional two-dimensional cultures. Overall, this pipeline provided evidence that lung PDTOs were effective in predicting patient drug response in vitro and suitable for designing novel therapeutic strategies [34]. Concomitantly, another group developed a protocol for the generation of lung organotypic cultures starting from both surgical resections and lung xenograft models [35]. Although the success rate was close to 88% in terms of organotypic growth initiation, the outgrowth of normal epithelial cells was observed in more than half of the short-term PDTOs. Thus, a deep histological and molecular annotation of the PDTOs and primitive tumors was performed to assess the generation of bona fide cancer models. Interestingly, only 15% of the PDTOs reached long-term growth, indicating that, in most cases, PDTOs were usable at the translational level only for a limited timeframe. On the other hand, short-term cultures exhibited extensive replication capability, a condition sufficient to perform different pharmacogenomic screenings, including innovative combination therapies [35]. The low success rate in stabilizing lung cancer PDTOs was ascribed to the rapid expansion of normal epithelial cells [36,37]. Sachs and colleagues addressed this issue by manually removing normal airway organoids from their cultures and by using nutlin-3a (MDM-2 inhibitor) to select neoplastic cells harboring *TP53* mutations [33]. Notably, a higher success rate was achieved starting from lung cancer malignant effusions. By using this approach, PDTOs confirmed their predictive value, as well as their potential to identify novel effective anti-cancer therapies directed against non-conventional biomarkers of lung cancer pathogenesis, such as *BRAF G464A* and *EGFR L747P* mutations [38]. An interesting work has recently compared different cell culture medium conditions to optimize the use of PDTOs in a co-clinical setting [39]. Specifically, the use of the airway organoid medium proposed by Sachs et al. showed a superior value in stabilizing tumor organoids. However, starting from 41 specimens, only three lung PDTOs were considered fully stabilized, as they were maintained in culture for more than 36 months. The molecular annotation of these three PDTOs was instructive in designing novel effective therapeutic strategies for rare genetic alterations [39]. In parallel, the first real-world study has recently successfully established 160 lung cancer PDTOs mainly from malignant serous effusions, with a success rate above 80%, indicating that this source of tumor cells was particularly productive [40].

#### 2.1.2. Major Limitations of Lung Cancer PDTOs

Overall, the stabilization rate for lung cancer organoids remains quite controversial, ranging from 7% to 80% [39,40], and significant discrepancies have been observed in distinct studies that have exploited the same growth medium conditions [39]. Many of the organotypic cultures reported in these works were short-term, and this aspect can influence the precise determination of the success rate. To date, the paucity of long-term stabilized organotypic cultures limits their use for functional validation studies, as well as the possibility of performing pharmacogenomic screening in a robust and reproducible manner. Since the main obstacle in obtaining pure lung cancer PDTOs is related to cross-contamination from the normal airway counterparts, surgical resections, as well as core biopsies, are particularly affected, as the total amount of cancer cells is limited in both cases [41]. Furthermore, methodological improvements are still necessary to optimize the cocktail of cytokines and growth factors to establish pure long-term cultures of lung cancer PTDOs [41].

### 2.2. The Translational Potential of Lung Cancer PDTOs

**Figure 2:**
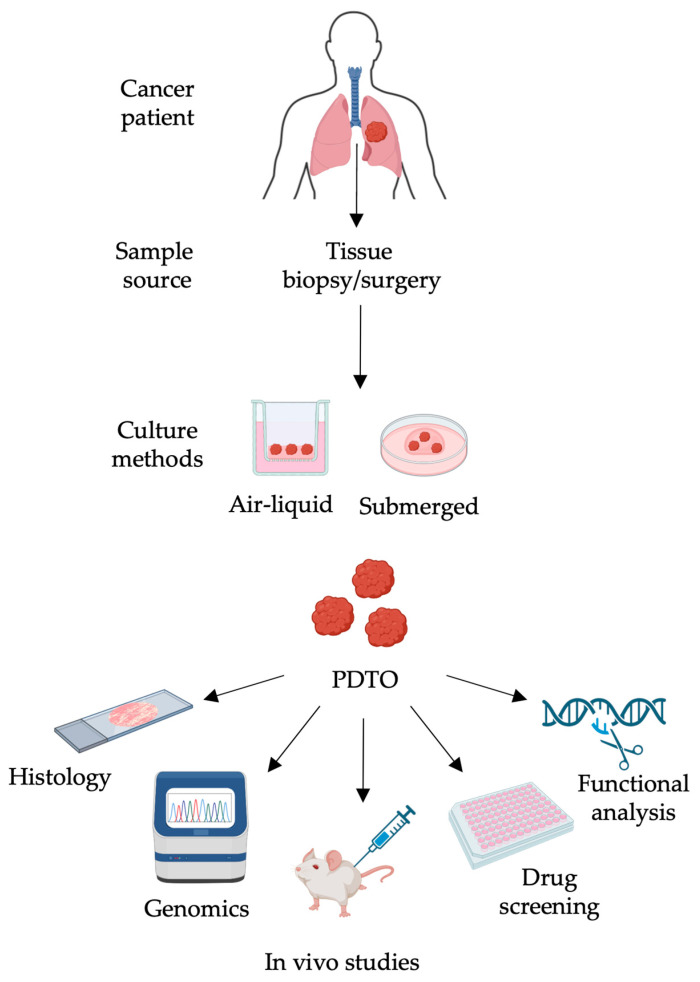
Advantages of patient-derived tumor organoids in a co-clinical setting. During the conventional clinical workflow, patient-derived tumor organoids (PDTOs) can be established from both surgical resections and core biopsies. PDTOs can be generated using both air-liquid interphase and submerged cell culture conditions. After histological and molecular validation, PTDOs might be used in a co-clinical setting to explore different therapeutic strategies, identifying the best treatment for each patient in both in vitro and in vivo scenarios. Moreover, genetically modified PDTOs can be used to identify the molecular circuits involved in drug resistance and consequently to design innovative strategies to overcome drug resistance in a personalized manner. Created with BioRender.com.

Remarkable observations of translational relevance have been demonstrated using bona fide PTDOs of oncogene-addicted NSCLC (Figure 2). The genomic analysis of lung tumors and matched organoids revealed the efficacy of olaparib in PTDOs expressing structural alterations of *BRCA2* of unknown significance, thus indicating the potential benefit of PARP inhibitors for a subset of lung cancer patients [34]. In an organotypic model of *FGFR1* amplification, typical of lung squamous cell carcinoma, a combination regimen based on the administration of MEK and FGFR inhibitors exhibited a strong synergistic activity both in vitro and in vivo compared to the administration of the FGFR inhibitor alone [35]. The potential predictive value of tumor organoids was also confirmed in PDTOs derived from NSCLC with rare genetic alterations [38]. A PDTO harboring the *EGFR L747P* mutation that confers gefitinib resistance showed susceptibility to the second-generation EGFR tyrosine kinase inhibitor (TKI) afatinib. In line with the PDTO response, the matched patient showed sensitivity to afatinib, as demonstrated by a progression-free survival of 9.5 months [38]. Moreover, in the same work, the efficacy of innovative treatments was tested in additional PDTOs. Indeed, poziotinib showed superior activity against the *ERBB2 G778_779insCPG* and *A775_G779insYVMA* alterations, while pralsetinib was particularly effective against the *CCDC6-RET* and *KIF5B–RET* rearrangements [38]. Furthermore, the generation of a PDTO after several lines of anti-EGFR TKIs revealed the maintenance of the original *EGFR^L858R^* alteration and the acquisition of co-mutations in *TP53* and *RB1*. In these tumor organoids, the primitive histology was maintained upon xenograft growth, and significant sensitivity to both survivin and Bcl-2 inhibitors (YM-155 and navitoclax, respectively) was observed [39]. Technological advances in the field have recently allowed the miniaturization of drug screening platforms using microwell array chips [42]. Specifically, the development of an integrated superhydrophobic microwell array chip showed that hundreds of PDTOs can be processed and immediately interrogated in terms of drug response curves within a week, highlighting the potential clinical impact of this pipeline. Interestingly, by using this platform, the rapid emergence of resistance to EGFR-TKI was detected in the tumor organoids, confirming the translational relevance of PDTOs [42]. The same platform was associated with an innovative algorithm of analysis to improve the prediction of drug sensitivity accuracy in high-throughput screening approaches [43]. In *ALK*-rearranged PDTOs, a multiparametric analysis including cancer stage and tumor cell proliferation rate showed that an *ALK*-directed drug response could be classified according to both the rearrangement status and the clinical data, highlighting its potential in the decision-making process [43]. More recently, a large study on lung cancer organoids mainly derived from serous effusions showed the predictive value of PDTOs for both chemotherapy and molecular targeted therapy regimens [40]. Functional validation analyses included 127 adenocarcinomas, 10 squamous cell carcinomas, 10 small cell lung cancer samples, 12 adenosquamous carcinomas, and one pulmonary sarcomatous carcinoma. Interestingly, in *ALK*-rearranged tumor organoids, the drug sensitivity tests perfectly matched the clinical response observed for both untreated and treated patients [40]. Furthermore, nine chemotherapy-treated patients and their matched PDTOs were analyzed and, also in this therapeutic setting, PTDOs showed their predictive value in terms of clinical response [40]. Overall, these data clearly show the translational relevance of lung cancer organoids in the decision-making process and in the design of next-generation clinical trials (Table 1).

## 3. Cancer Organoids for the Study of the Tumor Microenvironment

### 3.1. Components of the Tumor Microenvironment (TME)

**Figure 3:**
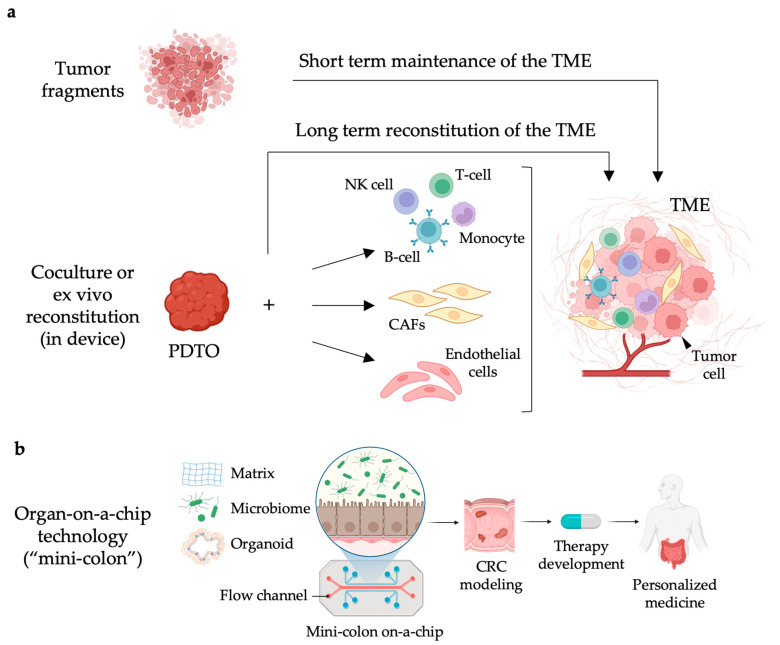
Patient-derived tumor organoids for next-generation functional precision immuno-oncology. (**a**) Starting from small tumor fragments and using specific cell culture conditions, it is possible to preserve in vitro different populations of the tumor microenvironment (TME), including immune cells, for short-term studies. Alternatively, using innovative bioengineered devices, an ex vivo reconstitution of PDTOs with specific subpopulations of the TME is also possible, including natural killer (NK) cells, B-cells, T-cells, monocytes, cancer-associated fibroblasts (CAFs), and endothelial cells, including the tumor vasculature. Both technologies are suitable to explore innovative immune therapy approaches in a personalized manner. (**b**) Recent advances in the bio-fabrication of microfluidic devices resulted in the generation of a functional “mini-colon” to recapitulate normal colon morphogenesis and to dynamically track, with unprecedented resolution, colorectal cancer (CRC) initiation and progression. The mini-organ technology represents the most-advanced pipeline for the implementation of PDTOs in a co-clinical setting for the design of more effective individualized therapeutic strategies. Created with BioRender.com.

The tumor microenvironment (TME) consists of several cellular populations, among which cancer-associated fibroblasts (CAFs), endothelial cells, and several immune cell types, including T and B lymphocytes, natural killer (NK) cells, tumor-associated macrophages (TAMs), and dendritic cells (DCs). The hierarchical architecture of tridimensional cultures, including the cell-cell communications established among different cell populations, makes organotypic cultures particularly attractive to investigate the crosstalk between the tumor and the related microenvironment [44] (Figure 3a).

As an example, in PDTOs of pancreatic ductal adenocarcinoma (PDAC), the production of WNT from CAFs was essential to sustain tumor organoid growth [45]. In a similar coculture setting of colorectal cancer organoids, CAFs were critical to promote the epithelial-to-mesenchymal transition, mimicking the aggressiveness of mesenchymal-like colon cancer [46]. The relevance of CAF–PDTO crosstalk in PDAC was also demonstrated by the tumor secretion of interleukin-1 (IL-1) and transforming growth factor-β (TGFβ), which negatively modulate CAF heterogeneity of pancreatic cancer [47].

### 3.2. Immune Organoids

#### 3.2.1. Immunotherapy and Cell Therapy Opportunities in Cancer Treatment

Another key component of the TME is represented by the infiltrating immune cells. Therefore, the possibility to generate immune organoids is particularly appealing at the translational level, especially considering the efficacy of immune therapies in several clinical studies. In particular, antibodies targeting immune checkpoints exhibited a clinical benefit in both advanced melanoma and NSCLC [48,49]. Alternative approaches include the use of the adoptive transfer of autologous tumor-infiltrating lymphocytes (TILs) upon ex vivo expansion, as demonstrated in melanoma [50]. Despite these encouraging results, only a fraction of patients responds to immunotherapy. On the other hand, the therapeutic potential of chimeric antigen receptor (CAR) T-cell therapy was successfully demonstrated in hematological malignancies, while their efficacy against solid tumors was particularly challenging for T-cell dysfunction and exhaustion [51]. Since several mechanisms of immune evasion can be engaged by tumor cells, the opportunity to faithfully mimic their crosstalk with the immune counterpart in vitro could be advantageous to identify in advance those patients who can benefit from immune checkpoint inhibition and/or cell therapy beyond conventional biomarkers, also defining potential mechanisms of resistance to this approach.

#### 3.2.2. Tumor-Reactive T-Cells and Cancer Organoid Cocultures

A pioneering protocol showed the possibility to generate tumor-reactive T-cells from autologous cocultures of peripheral blood mononuclear cells (PBMCs) and tumor organoids generated from surgical resections or core biopsies of colorectal and lung cancer specimens [52]. This pipeline required first the generation of bona fide tumor organoids that maintained MHC class I expression. Subsequently, to promote antigen presentation, tumor organoids were pre-stimulated overnight with IFNγ, a cytokine that in turn induces PD-L1, a negative modulator of T-cell activation. Then, tumor organoids were cocultured with peripheral T-cells in the presence of an anti-PD-1 blocking antibody in a dish coated with anti-CD28 to promote co-stimulation, while IL-2 was employed to sustain T-cell proliferation. After two weeks of coculture, fresh tumor organoids were re-challenged with the expanded tumor-reactive T-cells. IFNγ secretion and expression of the degranulation marker CD107a were used to measure specific T-cell reactivity against tumor organoids. As expected, no IFNγ secretion and CD107a were detected in the coculture of tumor-reactive T-cells with organoids obtained from normal tissue of the same patient, indicating that their reactivity was driven exclusively by tumor antigens. Accordingly, autologous T-cell killing was abrogated by adding an MHC class I-blocking antibody to the coculture [53]. Although tumor-reactive T-cells were generated only in a fraction of cocultures, this work indicates the potential use of peripheral T-cells to design a personalized adoptive cell therapy, exploiting the private nature of tumor neoantigens presented by matched tumor organoids. In an independent study, to determine the correlation between T-cell reactivity and the clinical response to neoadjuvant immune checkpoint inhibitors (ICIs) in an early-stage colorectal cancer clinical study, matched tumor organoids and T-cell cocultures were also interrogated [54]. Notably, the absence of tumor reactivity was observed in non-responders, while tumor-reactive T-cells were reported in only three out of six responders, indicating that this strategy could be suitable to study immune therapy resistance ex vivo, while its potential to correctly predict clinical response is limited to a fraction of patients. However, the ex vivo coculture analyses included only the use of an anti-PD-1 antibody, sparing the combination with the anti-CTLA 4 inhibitor that in contrast was tested in the corresponding patients. Consequently, more comprehensive analyses, including large cohorts of tumor organoids and matched reactive T-cells, will be fundamental to better clarify the predictive potential of this strategy in a co-clinical setting [54]. A different coculture protocol of PBMCs and matched PDTOs was successfully developed to isolate and expand tumor-reactive T-cells and to empirically identify tumor-targeting T-cell receptors (TCRs) against pancreatic cancer [55]. The analysis of immune checkpoints and matched ligands revealed patient heterogeneity, pointing to the relevance of interfering with patient-specific checkpoint regulators to enhance cytotoxic T-cell activity in a personalized manner. This approach also showed that TCRs selected from circulating lymphocytes of pancreatic patients matched those identified in tumor-infiltrating T lymphocytes (TILs), but rarely overlapped with those found in healthy individuals, indicating that pancreatic cancer patients can potentially benefit from autologous adoptive T-cell therapy alone or in combination with ICIs [55]. Moreover, the screening of patient-derived T-cells for personalized neoantigen recognition could greatly benefit from the use of PDTOs compared to traditional methods exploiting the antigen presentation functions of DCs. Indeed, PDTOs might reveal possible tumor-intrinsic defects such as a decrease in or total loss of HLA molecules, which prevent successful T-cell therapies [56].

#### 3.2.3. Tumor-Infiltrating T Lymphocytes (TILs) and Cancer Organoid Cocultures

A complementary approach to generate immune organoids requires the optimization of cocultures of TILs with matched PDTOs. In this case, tumors were mechanically dissociated and plated in a type I collagen matrix using air–liquid interphase (ALI) culture chambers. The non-enzymatic digestion of large tumor specimens from a wide cohort of surgically resected primary and metastatic tumors was critical to collect and preserve the tumor stroma, in particular CAFs, including the related immune counterpart [57]. This protocol was particularly effective to preserve different immune populations. Indeed, after two weeks of PDTO cultures originated from different tumor types, TILs, as well as macrophages, were maintained. Moreover, an immunophenotypic analysis also confirmed the presence of additional immune populations including B-cells, NK, and NKT cells, as well as the expression of the inhibitory receptor PD-1 on TILs [57]. Unfortunately, after one month of culture, a rapid decline of TILs was observed. This inexorable decrease was efficiently delayed by adding IL-2 to the culture conditions. Remarkably, the analysis at single-cell resolution of the primitive tumors and matched organoids at day 7 of culturing revealed the maintenance of different immune populations including T, B, and NK cells. Exhausted T-cells, T regulatory cells, and macrophages of the M2 phenotype were also present in immune PDTO cultures [57]. Furthermore, a single-cell analysis revealed that the TCR clonotypes were faithfully preserved in the matched PDTOs. Specifically, the V(D)J profiling of both TCR α and β chains of TILs showed a robust overlapping in the relative abundance of specific clonotypes between the PDTOs and the matched tumors. To functionalize immune organoid cocultures at the translational level, PDTOs were treated with anti-PD-1 within 7 days of culture to mimic a potential co-clinical timeframe compatible with the decision-making process. In a fraction of PDTOs from different tumor types, nivolumab triggered TIL activation, as demonstrated by the expression of IFNG, PRF1, and GZMB [57]. Unfortunately, an IHC analysis of PD-L1 expression on the matched tumors did not reveal a robust correlation between PD-L1 levels and TIL activation in the corresponding PDTOs, suggesting that, in line with what is observed in clinical practice, the PD-L1 level detected by IHC alone is not a robust biomarker of response to immune therapy. The predictive potential of TIL-mediated cytotoxicity against PDTOs was also observed in advanced rectal cancer patients [58].

#### 3.2.4. Short-Term TME Cancer Organoid Cocultures

Overall, the immune organoid “holistic” approach represents the ideal protocol when surgical resections are available and all the TME populations can be preserved and studied at the molecular and functional level in vitro. Unfortunately, the predictive potential in this context is limited by the short timeframe maintenance of immune cells in the coculture setting [57]. This protocol was successfully employed to evaluate the efficacy of cabozantinib and nivolumab in PDTOs of clear-cell renal cell carcinoma [59], and to test the efficacy of receptor inhibition by phosphatase recruitment (RIPR) as an innovative PD-1 inhibitor, which blocks both tonic and ligand-activated PD-1 signaling by inducing the cross-linking of PD-1 to CD45 [60]. More recently, ALI technology has been applied in PDTOs of basal cell carcinoma to show that TREM2+ skin cancer-associated macrophages are critical to sustain cancer cell growth through an immunosuppression-independent signaling pathway [61].

#### 3.2.5. CAR T-Cells and Cancer Organoid Cocultures

CAR T lymphocytes are extremely effective in hematological malignancies and represent a promising therapeutic strategy for the treatment of solid tumors as well [62]. In this context, PDTO cocultures represent robust preclinical models to test various immunotherapy approaches in a personalized manner, including CAR T efficacy, on-target/off-tumor T-cell reactivity, and mechanisms of resistance to cell therapy. In colorectal PTDOs, CAR-engineered NK-92 cells against a subset of tumor-associated antigens resulted in a cytotoxic response toward normal tissue [63], showing the relevance of this approach in predicting the off-target effects of CARs. In glioblastoma, an optimized protocol to generate a robust biobank of PTDO models has been recently established. This platform preserved glioblastoma tumor heterogeneity at both the genomic and transcriptomic level, including the original aggressiveness when re-implanted in vivo. Most importantly, a detailed methodology to evaluate CAR T killing efficacy and target specificity was provided, indicating the potential of this platform at the translational level [64]. The effectiveness of CAR T therapy was also demonstrated in bladder cancer PDTOs. In this coculture setting, specific targeting was observed against MUC-positive PDTOs, while no cytotoxicity was detected in MUC-negative tridimensional cultures [65]. Moreover, PDTO cocultures are also suitable for evaluating dual-agent therapy efficacy. Indeed, the combination of CAR T-cells with birinapant, a proapoptotic agent, exhibited significantly synergistic activity in HER2+ colorectal tumor organoids, while anti-HER2 CAR T-cells alone were only minimally effective [66].

### 3.3. Innovative Tools to Study the Interplay between Cancer Organoids and the TME

#### 3.3.1. Imaging Technologies

To optimize immune-directed therapy approaches in a personalized manner, a 3D imaging transcriptomic platform was developed and tested in dozens of human cancer organoids in parallel. The pipeline was interrogated to dynamically analyze the efficacy of different immunotherapy strategies such as CAR, conventional T-cell receptor therapies, and genetically modified αβ T-cells to express a γδ TCR. By comparing and correlating T-cell activity over time in PDTO cocultures, killing efficacy and persistence were defined for each cell therapy strategy, highlighting the robustness of this pipeline to select the optimal T-cell therapy for each patient [67]. Moreover, a multispectral 3D live organoid imaging platform has been recently developed and tested in PDTOs to improve fluorescence-guided surgery (FGS), a procedure consisting of fluorescent dyes and a near-infrared camera that allows surgeons to analyze tumor tissue in real time to obtain a complete tumor resection. This pipeline was successfully tested in different preclinical models of tumors, defining specific probes to identify metastatic lesions [68].

#### 3.3.2. Microfluidic Technologies

Simultaneously, technological advances in microfluidic devices have improved the capability of better mimicking in vitro the tumor and TME interplay [69]. A pioneering technology included three different channels, in which the central one was loaded with collagen embedding the tumor fragments for PDTO generation, while the two lateral ones were infused with medium. Interestingly, these microfluidic patient-derived organotypic tumor spheroids preserved the original immune population in short-term cultures, making the device particularly relevant to test immune therapy regimens. Specifically, ICI response, immune cells, and PDTO interactions, as well as cytokine profiling, could be directly analyzed ex vivo, showing the potential impact of this technology in next-generation immune precision oncology [69]. This device has been recently exploited to validate TANK-binding kinase 1 (TBK1) as a new immune evasion gene. By interrogating microfluidic devices, TBK1 inhibition enhanced the efficacy of anti-PD-1 therapy in patient-derived organotypic tumor spheroids, showing that TBK1 is a novel potential target to overcome immunotherapy resistance [70]. Further biotechnological advances resulted in the generation of vascularized microfluidic devices. This technology was efficiently employed to mimic the brain tumor vasculature of glioblastoma and to test the efficacy of targeted nanotherapeutics. Indeed, by recreating in vitro the blood–brain barrier (BBB), it was possible to evaluate the efficacy of modified nanoparticles to increase BBB permeability and thus deliver cisplatin. This work clearly demonstrated the relevance of this system to accurately reproduce the brain vasculature, to study its permeability, and to predict the therapeutic efficacy of innovative nanoparticle formulation in an ex vivo setting [71]. Additional improvements allowed the generation of perfusable microvessel devices, where endothelial cells, after a first phase of adhesion and spreading, were subjected to a constant flow to generate physiological shear stress. This technology was exploited to study the process of mosaic vessel formation, consisting of both endothelial and tumor cell structures as vessel pull and vessel constriction, also dissecting the process of metastatization engaged by circulating tumor cells. Interestingly, tumor organoids exhibited a more pronounced growth rate when maintained under perfusion, while tridimensional cultures failed to grow over 8 days without perfusion. Overall, these tools are extremely effective for dynamically tracking and deeply characterizing the crosstalk between tumor and endothelial cells, including the process of tumor dissemination in an ex vivo setting [72].

#### 3.3.3. Organ-on-a-Chip Technologies

Moreover, the improvement in microfabrication and biotechnological tissue engineering resulted in the development of a “mini-colon” on-a-chip (Figure 3b). Specifically, a biocompatible perfusion scaffold was established to faithfully recapitulate the intestinal crypt and villus architecture. This device preserved key topological conditions of the colon that favor intestinal stem cell self-organization, commitment, and differentiation until the reproduction in vitro of a functional “mini-colon”. The device maintained the features of the original organ such as cellular heterogeneity, tissue architecture, and regeneration capability, making the device extremely attractive for disease modeling [73]. Recently, using genetically modified murine colon cells, where the activation of the oncogenic mutations was spatiotemporally modulated only in a small fraction of cells, the mini-colon system dynamically reproduced at an unprecedented resolution the process of tumor initiation and development from a healthy murine epithelial architecture. The resulting model of colorectal cancer faithfully recapitulated context-dependent tumor plasticity, intra-tumoral and inter-tumoral heterogeneity, and the pathophysiological hallmarks characterizing colorectal cancer. The flexibility of the device was instrumental to screen environmental factors that, by entering into contact with the luminal colonocytes, could influence colorectal cancer pathogenesis and fitness, and to study microbiota-derived metabolites and the impact of diet in pathophysiological responses to procaryote metabolism [74]. Notably, the mini-colon organ-on-a-chip technology was successfully implemented to mimic, in a topobiologically healthy human colon epithelium, the process of human colorectal cancer (CRC) initiation and progression. By including a negligible fraction of GFP-labeled CRC cells derived from tumor biopsies in a suspension of human healthy colon cells, the emergence of hyperplastic structures from the healthy epithelium was monitored over time until the development of a full-blown tumor. Most importantly, the integration of CAFs in the mini-colon device showed that CAF-dependent IL-1β release was pivotal to trigger a proinflammatory program in CRC, a process responsible for MMP7-mediated CRC dissemination. Moreover, in the microsatellite-unstable (MSI) CRC mini-colon where both CAFs and TILs were included, CAFs suppressed T-cell anti-tumor reactivity by promoting the expression of PD-L1. Remarkably, the addition of atezolizumab restored TIL activity also in the presence of CAFs in MSI but not in the microsatellite-stable (MSS) CRC mini-colons [75], clearly demonstrating the superior value of the “mini-colon” device in precision colorectal cancer immunotherapy in a co-clinical setting.

## 4. Conclusions

Tumor relapse after surgical resection, as well as resistance to chemoimmunotherapy or molecular targeted strategies, represent the main roadblocks for cancer eradication. Although precision oncology has dramatically improved patient stratification and outcomes, as epitomized in NSCLC, increasing evidence supports a more complex scenario where genetic heterogeneity, tumor cell plasticity, and the crosstalk with the microenvironment all contribute to cancer resilience to treatments.

### 4.1. Achievements

Given the recent biotechnological advances in ameliorating cell culture conditions and in the microfabrication of perfusable devices, the generation and characterization of patient-derived tumor organoids and immune organoids is theoretically possible in a personalized manner. The current technology clearly shows the tremendous advantage of including PDTOs in the clinical decision-making process. Promising results have been already obtained by interrogating CRC-derived PDTOs, as demonstrated by their high predictive value for diverse standard-of-care regimens [76].

### 4.2. Limitations

The most critical limitations of this approach include the low efficiency of organoid establishment from specific tumor types and the relatively short lifespan of immune-PDTO cultures. Therefore, additional refinements in cell culture conditions, as well as the development of more advanced perfusable devices, are mandatory to improve immune PDTO generation efficiency and to support their long-term viability in ex vivo conditions.

### 4.3. Future Directions

To implement PDTOs into clinical practice, it will be necessary to establish robust workflows from bed to benchside and back to patients, an effort involving several specialists in different disciplines, including oncologists, radiologists, pathologists, biologists, and engineers, as well as stakeholders and regulatory agencies. Although this scenario could be considered still utopistic, the scientific community aims at concretizing the inclusion of PDTOs, immune PTDOs, and PDTO mini-organs in next-generation functional precision oncology [77,78].

## Figures and Tables

**Table 1:** Pre-clinical studies on lung cancer PDTOs. The most effective targeted treatment is reported for each oncogenic driver alteration investigated in the selected works. (*) Response confirmed in the matched patient. If not indicated, patient-related information is not reported in the study. (**) Only data from PDTO drug screening with an IC50 of less than 1 μM are included.

NSCLC Subtype	PDTO		Reference
	Genetic Alteration	Effective Treatment	
Adenocarcinoma	BRCA2 p.W2619C	olaparib	Kim et al., 2019 [34]
Adenocarcinoma	EGFR p.L858R	erlotinib	
Adenocarcinoma	EGFR p.L858R, MET amplification	crizotinib	
Adenocarcinoma	EFGR exon 19 del	erlotinib	Shi et al., 2020 [35]
Adenocarcinoma	KRAS G13C, KRAS amplification	trametinib	
Squamous cell carcinoma	FGFR1 amplification	BGJ398 + trametinib	
Adenocarcinoma	EGFR exon 19 del, EGFR T790M, BRAF V600E, BRAF G464A, EGFR C797S	dabrafenib + trametinib	Kim et al., 2021 [38]
Adenocarcinoma	EGFR L747P	afatinib *	
Adenocarcinoma	ERBB2 G778_779insCPG	poziotinib	
Adenocarcinoma	ERBB2 A775_G779insYVMA	poziotinib	
Adenocarcinoma	CCDC6-RET	pralsetinib	
Adenocarcinoma	KIF5B-RET	pralsetinib	
Adenocarcinoma	BRAF G469A	trametinib + erlotinib	Yokota et al., 2021 [39]
Adenocarcinoma	TPM3-ROS1 fusion	entrectinib	
Adenocarcinoma	EGFR L858R	navitoclax + YM-155	
Adenocarcinoma	EGFR L858R, T790M	osimertinib *	Wang et al., 2023 [40] **
Adenocarcinoma	EGFR exon 19 del, T790M	osimertinib *	
Adenocarcinoma	EGFR exon 19 del	osimertinib *	
Adenocarcinoma	EML4-ALK fusion, ALK-C17orf75 fusion	alectinib *	
Adenocarcinoma	EML4-ALK fusion	alectinib *	
Adenocarcinoma	ERBB2 exon 20 ins	pyrotinib *	
Adenocarcinoma	EGFR L858R, MET amplification	osimertinib + savolitinib *	
Adenocarcinoma	EGFR exon 19 del, T790M, RET fusion	osimertinib + cabozantinib *	
Adenocarcinoma	EGFR exon 19 indel	gefitinib	Hu et al., 2021 [42] **
Adenocarcinoma	EGFR L858R	gefitinib	
Adenocarcinoma	EGFR G719A	gefitinib	
Adenocarcinoma	EML4-ALK (E6-A20)	crizotinib	
